# Recent progress in fluorescent chemosensors for selective aldehyde detection

**DOI:** 10.1039/d5ra01010a

**Published:** 2025-04-01

**Authors:** Keshav Semwal, Avijit Kumar Das

**Affiliations:** a Department of Chemistry, Christ University Hosur Road Bangalore 560029 Karnataka India avijitkumar.das@christuniversity.in

## Abstract

Aldehydes (R-CHO) are volatile and chemically active compounds prevalent in the environment, industrial chemicals, food fermentation, and human metabolism. Their release into the atmosphere can lead to pollution and adverse health effects, from mild irritation to severe respiratory and immune system damage. Among aldehydes, formaldehyde (FA) and acetaldehyde (AA) are notable indoor air pollutants with stringent safety limits set by organizations like WHO and OSHA. Current detection techniques, including gas and liquid chromatography, while highly accurate, are expensive and require trained personnel. Conventional sensors, such as semiconductor and chemiresistive types, offer portability and online detection but are hindered by poor selectivity and sensitivity. Optical chemosensors, which operate based on fluorescence or colorimetric changes induced by chemical interactions, have emerged as a promising alternative due to their high sensitivity, selectivity, cost-effectiveness, and portability. This review explores the advancements in optical chemosensors for aldehyde detection, emphasizing novel molecular designs utilizing mechanisms like imine bond formation, cyclization reactions, and aza-Cope rearrangements *etc.* The applications of these sensors in environmental monitoring, biomedical analysis, and other industries are highlighted, showcasing their potential for real-time, low-concentration detection of aldehydes in diverse settings.

## Abstract 2

Aldehydes (R-CHO) are reactive compounds in nature, industry, food, and metabolism, posing pollution and health risks. This review covers advances in optical chemosensors for aldehyde detection, highlighting novel designs and key sensing mechanisms.

## Graphical abstract

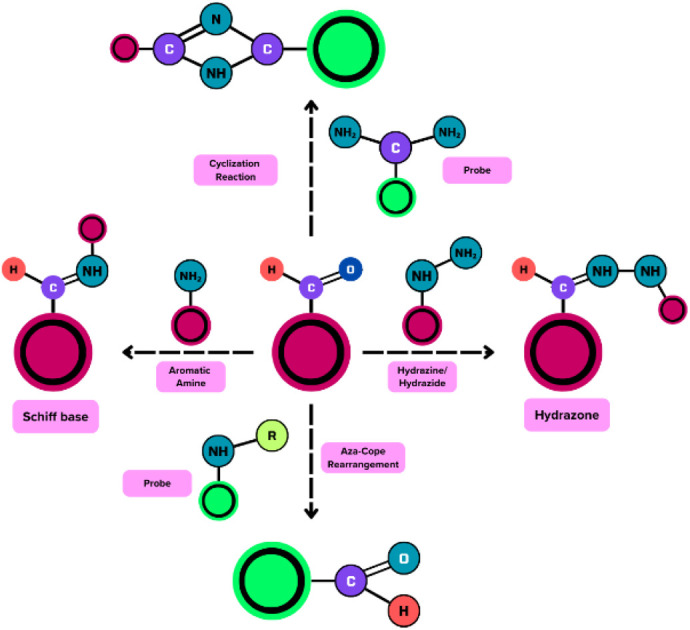

## 1 Introduction

Aldehydes (R-CHO) are volatile, chemically active compounds found widely in the environment, industrial chemicals, food fermentation, and human metabolites.^1^ Their release into the air can cause pollution and health issues, ranging from skin and eye irritation to severe respiratory and immune system damage upon exposure.^2^ Volatile aldehydes, present in exhaled breath, are key biomarkers for cancers like lung cancer, driving research on noninvasive disease diagnosis through breath analysis of aldehydes and other volatile organic compounds (VOCs).^3^

Among all aldehydes, formaldehyde (FA) and acetaldehyde (AA), the smallest and most volatile aldehydes, are common indoor air pollutants posing significant health risks. As a result, they are widely studied as target analytes in chemosensor development, particularly for gas-phase detection. The World Health Organization (WHO) recommends a safe indoor FA exposure limit of 80 ppb over 30 minutes, while the Occupational Safety and Health Administration (OSHA) sets it at 750 ppb.^4^ For AA, WHO's limit is 5 ppb, and OSHA's permissible level is 200 ppm.^5^

Therefore, there is a continuous demand to develop sensors for aldehyde detection with lower concentration limits for applications in various fields like environmental monitoring, biomedical analysis, pharmaceuticals, and food industries.^6^ Various techniques such as gas chromatography, and high-performance liquid chromatography have been used to measure aldehydes but these methods are expensive, complex, and require trained operators.^7^ On the other hand, easier-to-use sensors, such as semiconductor films, surface acoustic waves, and chemiresistive sensors, offer online detection but suffer from poor selectivity and sensitivity.^8^ Electrochemical sensors are widely used in liquid phase detection, but adapting them to gas phase detection remains challenging due to issues with collecting and concentrating gaseous analytes. Research into all-solid-phase electrochemical sensors aims to improve gas detection, but these still face limitations due to low analyte absorption and low ionic conductivity. To address these issues, increasing efforts are being made to develop optical chemosensors that offer high sensitivity, selectivity, low cost, and portability for real-time aldehyde detection.^9^ Optical chemosensors detect aldehydes through color changes or fluorescence modulation (intensity or wavelength shift) triggered by chemical interactions, such as aldimine condensation. Both colorimetric and fluorometric sensors, including dual-mode sensors, are effective for gas-phase aldehyde detection, benefiting from molecular design flexibility that enhances sensitivity, selectivity, and system miniaturization.^10^ In some cases, colorimetric sensors allow detection by the naked eye, though typically only at ppm–ppb detection limits. Few of the processes involved in the detection mechanism are PET, ICT and ESPIT. PET is a quenching pathway, involving the migration of electron between a photoexcited and ground state molecule due to absorption of light, creating an electron donor and an electron acceptor.^11^ In ICT upon photoexcitation of the molecules, charge transfer from the electron donor to the electron acceptor, this changes the electron effects within the fluorophore, leading to change in the spectrum (blue/red shift). On the other hand, ESIPT molecules have two different modes of emission, the enol form causes short wavelength emission and the keto form causes longer wavelength emission. The fluorescence properties can be adjusted by changing the environmental properties like electron donor and acceptor, pH of the solvent, *etc.*^12^ The current unresolved challenges include the need for stoichiometric amounts of chemosensors to generate a signal, which may also disrupt the analyte's environment. Therefore, reversible chemosensors are highly desirable.^13^ The major step in this field of research is the development of near-infrared (NIR) probes for feasible detection.^14^ In this review we have focused various optical chemosensors for aldehyde detection focusing enlisting the designed material by utilizing various fluorescent molecules with different unique mechanism like imine bond formation, cyclization reaction, aza-Cope rearrangement *etc.* with their various application.

## 2 Detection by amine

The detection of aldehydes using amines is a widely studied area due to its relevance environmental and biological monitoring. Nucleophilic amines react with aldehydes to form stable products, enabling their detection and quantification.^15^ Aromatic amines react with aldehydes to form Schiff bases.^16^ These Schiff bases exhibit distinct fluorescence properties for detection of various biological species. The stability of imine-based sensors depends on the structural design, where electron-withdrawing groups, conjugation, and intramolecular hydrogen bonding enhance resistance to hydrolysis. Selectivity is tuned by modifying substituents to create specific noncovalent interactions, such as hydrogen bonding and π–π interactions, improving binding affinity toward target analytes.^17^ Hydrazine and its derivative hydrazide react with aldehydes to give hydrazone derivatives.^18^ These derivatives have distinctive physical and chemical properties, which can be used for quantitative and qualitative analysis.^19^ A hydrazine or hydrazide group can react with an aldehyde or ketone, resulting in the formation of a dehydration product known as a hydrazone. This compound is a type of Schiff base characterized by a double bond between the carbon atom of the original carbonyl group and the terminal hydrazino nitrogen. Compared to a standard imine formed between an amine and a carbonyl group, a hydrazone is more stable and less prone to hydrolysis, making it less likely to revert to its starting materials.^20^

### 2.1 Detection by aromatic amine

**Fig. 1:**
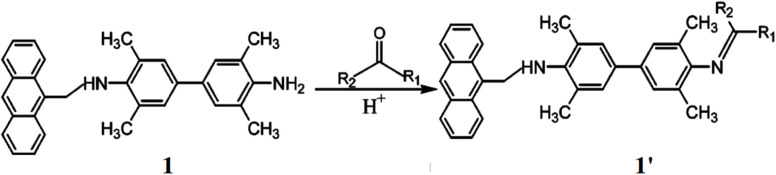
The structure of ligand 1 and the sensing mechanism with carbonyl compounds (reproduced from ref. 21 with permission from American Chemical Society, copyright 2003).

Yang *et al.* presented a probe for the detection of aliphatic aldehydes by reversible reaction mechanism between 3,3′,5,5′-tetramethyl-*N*-(9-anthrylmethyl)benzidine (TMAB, 1) and aliphatic aldehydes.^21^ TMAB interacts with aliphatic aldehydes to form a Schiff base (1′). The lone pair electrons of the amino groups transfer to methylanthracene and TMB, quenching of the fluorescence. Upon protonation or Schiff base formation, the electron transfer is inhibited, resulting in enhanced fluorescence of both fluorophores (Fig. 1). The maximum excitation wavelengths for anthracene and TMB are 296 nm and 368 nm, respectively, while both emit fluorescence at 410 nm. A significant fluorescence enhancement was observed by 8.3-fold for TMB, while anthracene showed only 1.5-fold. The detection limit of the probe 1 towards aliphatic aldehydes was determined to be 0.003. The response of 1 to aldehydes and ketones followed the decreasing order: *n*-butyraldehyde > propionaldehyde > isobutyraldehyde > glutaraldehyde > acetaldehyde > formaldehyde > ethyl methyl ketone > diethyl ketone > acetone. Additionally, the fluorescence of 1 was found to be independent of pH.

**Fig. 2:**
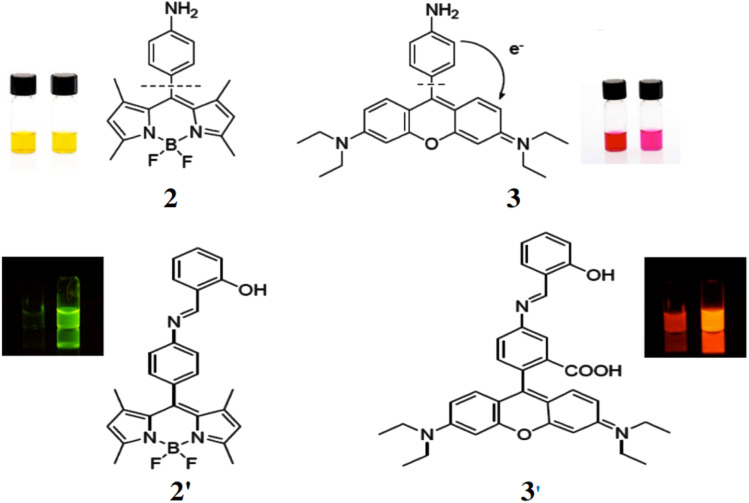
(Top) Structures of amine (2) and xanthene derivatives (3). (Bottom) Structures of imine derivatives of 2 (**2′**) and 3 (**3′**) (Reproduced from ref. 22 with permission from MDPI, copyright 2016).

Bane *et al.* reported two dyes based on a photoinduced electron transfer (PET) mechanism for quenching of fluorescence, specifically for the detection of aldehydes.^22^ Amino derivatives of a boron dipyrromethene (2) and a xanthene-derived fluorophore (3) were synthesized. Both compounds are nearly non-fluorescent in polar and apolar solvents but generate fluorescent imine derivatives (2′ and 3′) upon reacting with salicylaldehyde (Fig. 2). This reaction increases the fluorescence quantum yield by nearly tenfold, from 0.05 to 0.4. These dyes serve as highly useful tools for the selective fluorescent detection of various aldehydes.

**Fig. 3:**
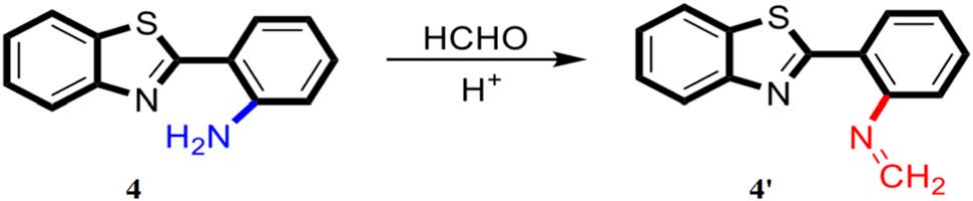
The structure of ligand 4 and probable sensing mechanism of formaldehyde by the probe (reproduced from ref. 23 with permission from American Chemical Society, copyright 2023).

Ding *et al.* reported a benzothiazole-based fluorescent probe 4 containing an amino group at the ortho-position exhibiting a significant fluorescence response in the presence of formaldehyde under acidic conditions, due to the formation of an imine (4′) (Fig. 3).^23^ The probe 4 exhibits intramolecular hydrogen bonding between the amino NH group and the benzothiazole N, leading to an ESIPT, which is responsible for the fluorescence. As the concentration of formaldehyde increases, the fluorescence intensity at 455 nm decreases sharply. The detection limit was determined to be approximately 16.6 μM for formaldehyde, and the limit of quantification was found to be 55.3 μM. The probe generates little to no fluorescence, making it useful for distinguishing between aldehydes and ketones. It can be dissolved in aqueous solvents of varying pH to create a sensor array, which is capable of detecting seven different aldehydes (formaldehyde, glyoxal, propionaldehyde, acrolein, hexanal, dodecyl aldehyde, and valeraldehyde) in samples. The probe can also be applied to test paper for the visual detection of formaldehyde vapors.

**Fig. 4:**
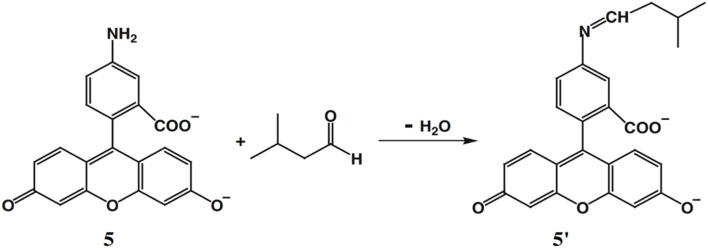
The structure of ligand 5 and aldehyde sensing pathway (reproduced from ref. 24 with permission from Springer, copyright 2011).

Wei *et al.* developed a probe 5 composed of 5-aminofluorescein for selective detection of aldehydes with high sensitivity, distinguishing them from other carbonyl compounds such as ketones and acids, unlike hydrazine reagents.^24^ This probe 5 can be used for monitoring microbial oxidation from primary alcohols to aldehydes during the biotransformation by *Gluconobacter oxydans*. The ligand 5 contains an amino group with weak fluorescence, which reacts with aldehydes to form an imine (5′) with strong fluorescence at 538 nm (*λ*_ex_ = 485 nm) (Fig. 4). The probe 5 demonstrated a good response in neutral and weakly alkaline conditions (pH 6.5–8.0), with a detection limit of 6.94 nM.

**Fig. 5:**
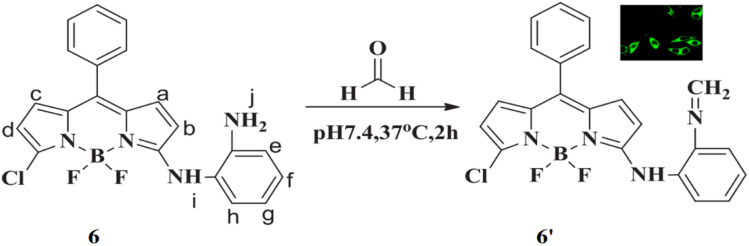
Chemical structure of 6 and the mechanism of reaction of 6 with formaldehyde and Confocal microscopy fluorescence imaging of exogenous in HeLa cells (reproduced from ref. 25 with permission from Elsevier, copyright 2018).

Guo *et al.* reported a fluorescent probe 6 for detecting formaldehyde comprising boron dipyrromethene as a fluorophore and *o*-phenylenediamine (OPDA) as the reacting group.^25^ The absorption and emission maxima of the probe are 482 nm and 525 nm, respectively, with a quantum yield of 0.016 ± 0.0022. The amino group in the probe 6 reacts with formaldehyde by the formation of 6′, causing a shift in the fluorescence band from 525 nm to 548 nm, resulting in a significant increase in intensity with strong green fluorescence (Fig. 5). Upon reaction with formaldehyde, the fluorescence intensity increases 12-fold with a high quantum yield of 0.252 ± 0.0310. The detection limit of 6 for formaldehyde was determined to be 0.104 μM. The sensor 6 is highly selective for formaldehyde, distinguishing it from other aldehydes and ions in both aqueous solutions and living cells. Thus, the probe 6 enables tracking of both exogenous and endogenous formaldehyde in living cells as well as gaseous formaldehyde.

**Fig. 6:**
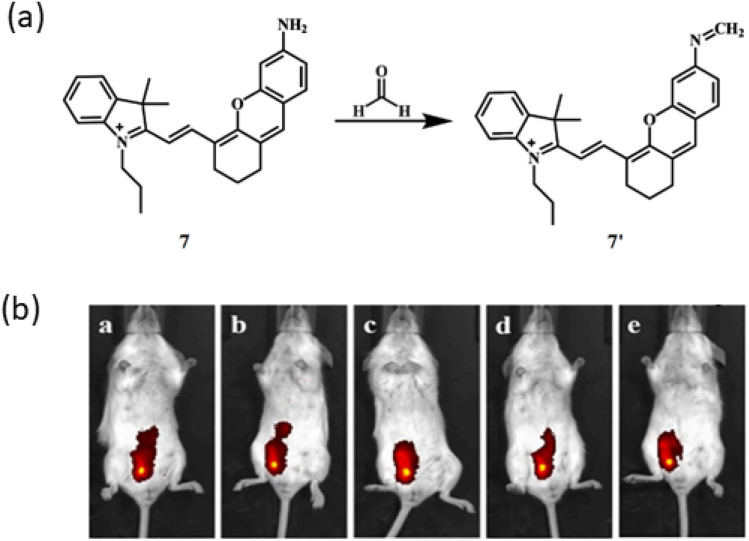
(a) Structure of ligand 7 and reaction of 7 with FA. (b) Fluorescence imaging of BALB/c mice by ligand 7 with FA at different times (0, 10, 20, 30 min) (reproduced from ref. 26 with permission from Elsevier, copyright 2020).

Yang *et al.* developed a near-infrared fluorescence probe 7 for detection of formaldehyde based on hemicyanine skeleton utilizing Schiff base reaction with an emission at 708 nm.^26^ The reaction is based on Schiff base reaction and amine 7 reacts with the aldehyde to give an imine (7′) exhibiting absorption and emission maxima at 670 nm and 708 nm respectively with a detection limit at 1.87 μmol L^−1^ by a response time of 30 minutes (Fig. 6). The probe 7 can be used for developing paper chips, which can be used for real time detection of FA for the detection of food samples and detection of endogenous FA in mice.

### 2.2 Detection by hydrazine

**Fig. 7:**
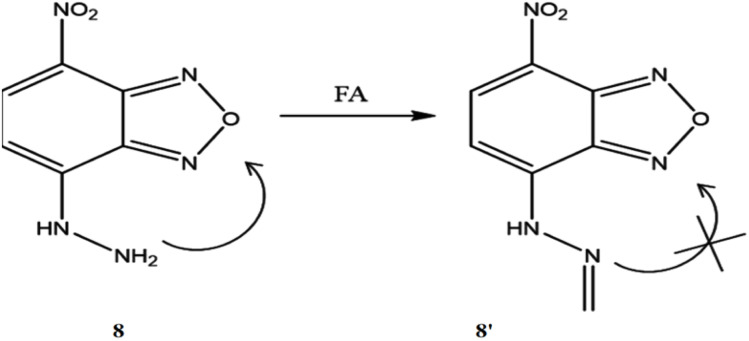
The structure of ligand 8 and detection pathway of formaldehyde by 8 (reproduced from ref. 27 with permission from Elsevier, copyright 2020).

Wang *et al.* developed a probe (8) to exhibit a direct fluorescence enhancement response to formaldehyde.^27^ The hydrazine group of 8 functioned as a fluorescence quencher *via* PET mechanism for the selective recognition of formaldehyde. This recognition occurred through the formation of a Schiff base compound *via* an aldimine condensation reaction inhibiting the PET pathway and activated the fluorescence of the probe (8′) (Fig. 7). The probe 8 displayed a strong fluorescence response to formaldehyde under strongly acidic conditions but was insensitive to formaldehyde under less acidic medium. Initially, the probe exhibited weak fluorescence at 550 nm, which was increased in the presence of formaldehyde significantly. The detection limit of 8 towards formaldehyde was determined to be 0.89 μg L^−1^, with a linear detection range of 0.015–0.8 mg L^−1^.

**Fig. 8:**
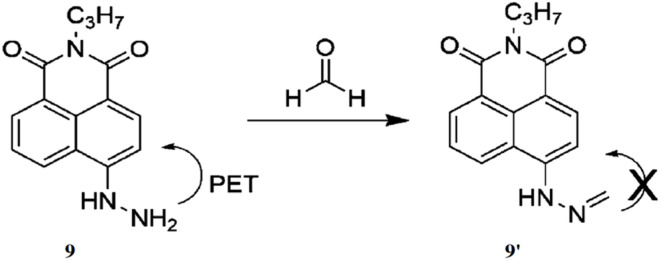
The chemical structure of 9 and sensing pathway of formaldehyde (reproduced from ref. 28 with permission from wiley-VCH, copyright 2016).

Lin *et al.* presented a novel two-photon fluorescent probe (9) for detecting formaldehyde (FA) in living tissues.^28^ The probe 9 consists of a 1,8-naphthalimide, a two-photon dye scaffold, with a hydrazine moiety incorporated, which specifically reacts with formaldehyde. This reaction turns off the photoinduced electron transfer (PET) mechanism, resulting in the increase of fluorescence by the formation of 9′ (Fig. 8). The probe 9 exhibited a strong fluorescence peak around 543 nm, with an immediate 325-fold increase in fluorescence, which further increased by 900-fold after 30 minutes of incubation. The detection limit of 9 towards formaldehyde was demonstrated as 7.1 × 10^−7^ M. The probe 9 exhibited high selectivity for formaldehyde over other biological species, along with minimal toxicity and photostability.

**Fig. 9:**
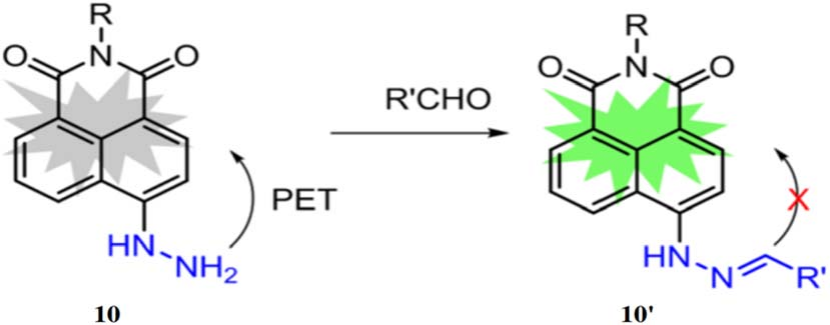
Chemical structure of ligand 10 and probable sensing mechanism with aldehyde (reproduced from ref. 29 with permission from American Chemical Society, copyright 2022).

A lipophilic naphthalimide hydrazine-based fluorescent probe (10) was introduced for profiling aldehydes generated during the oxidation of unsaturated lipids.^29^ Dodecyl amine was incorporated to enhance the probe's lipophilicity and surfactant properties, enabling effective aldehyde detection (Fig. 9). The probe 10 exhibited distinctive fluorescence responses, showing a 40-fold increase in fluorescence with malondialdehyde (MDA) and a 25-fold increase with hexanal, appearing blue and green, respectively. The probe's maximum excitation and emission wavelengths were 450 nm and 530 nm, which was shifted to 350 nm and 450 nm respectively with MDA. The probe 10 demonstrated the ability to detect eight major oxidation products, including seven aldehydes such as formaldehyde, acetaldehyde, hexanal, malondialdehyde, 4-hydroxy-2-nonenal, 9-oxononanoic acid, and 2-propyloxirane.

**Fig. 10:**
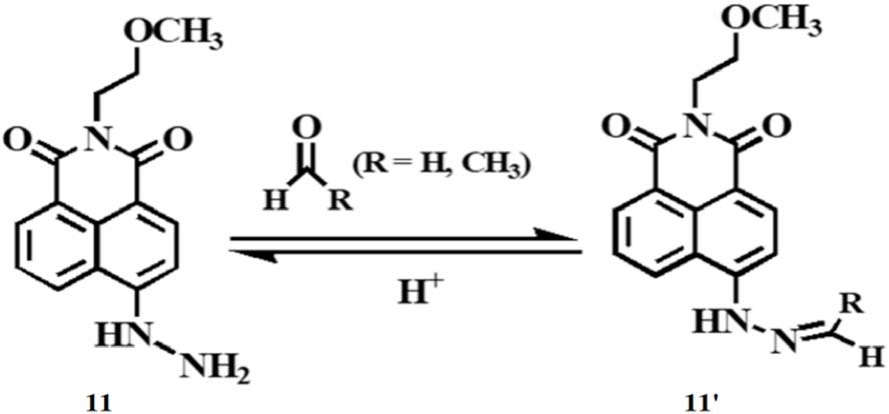
The chemical structure of 11 and nucleophilic addition reaction between the probe 11 and aldehydes (reproduced from ref. 30 with permission from American Chemical Society, copyright 2021).

Wang *et al.* developed a fluorescent sensing film for detecting formaldehyde and acetaldehyde using a naphthalimide functionalized penetrated into SiO_2_ inverse opal photonic crystals (11).^30^ The naphthalimide reacts with formaldehyde and acetaldehyde *via* nucleophilic addition by the formation of 11′, producing a strong fluorescence emission at 550 nm for formaldehyde and 553 nm for acetaldehyde (Fig. 10). The fluorescence intensities of 11 were enhanced by 13.8-fold for formaldehyde and 26.3-fold for acetaldehyde. The inverse opal photonic crystals amplify fluorescence through the “slow photon effect”, resulting in a threefold increase in detection sensitivity. Additionally, the photonic crystals accelerate aldehyde diffusion and provide ample reaction sites, enabling a rapid response time of just 1 minute. The detection limits of 11 were determined to be 10.6 nM for formaldehyde and 7.3 nM for acetaldehyde. The sensing film 11 can be regenerated by immersion in an acidic aqueous solution and is suitable for detecting formaldehyde and acetaldehyde in air, aqueous, and living systems.

**Fig. 11:**
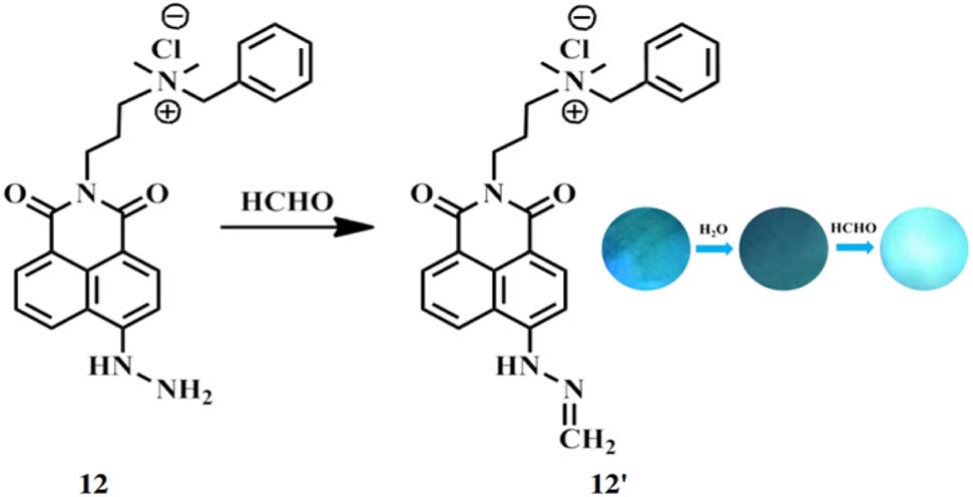
Mechanism of aldehyde sensing by 12 and photographs of the membrane (reproduced from ref. 31 with permission from American Chemical Society, copyright 2020).

Gao *et al.* reported a membrane (12) comprising ANH attached to cellulose nanocrystals (CNCs) to create a chiral photonic membrane with high sensitivity for detecting formaldehyde.^31^ The CNC surfaces contain numerous anionic sulfate groups, allowing easy attachment of the cationic ANH molecules. The hydrazine group in ANH reacts with formaldehyde, producing 12′ with a “turn-on” fluorescent signal (Fig. 11). The membrane 12 exhibits a noticeable color change even at low formaldehyde concentrations ranging from 0.438 to 2.103 ppm. This membrane 12 is suitable for detecting trace levels of formaldehyde in living samples and atmospheres.

**Fig. 12:**
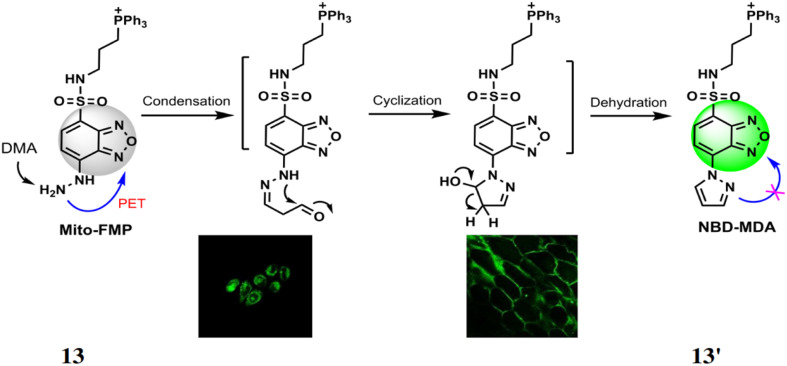
The proposed reaction of probe 13 with MDA and detection of MDA in HeLa cells and onion tissues (reproduced from ref. 32 with permission from Royal Society of Chemistry, copyright 2017).

Lin *et al.* synthesized a probe 13 for detection of malondialdehyde (MDA) based on benzoxadiazole chromophore, triphenylphosphonium as the mitochondrial targeting site and hydrazine as reacting site.^32^ The probe reacts with MDA to yield a. Initially, the free probe 13 is almost non-fluorescent with excitation at 373 nm and quantum yield of 0.0015 but upon reaction with MDA led to the formation of 13′ which inhibits PET to enhance fluorescence intensity by 774-fold at 554 nm (Fig. 12). The detection limit was determined to be 4.54 × 10^−7^ M. The probe 13 functions efficiently in neutral and alkaline environments, making it compatible with physiological conditions showing high selectivity for MDA over other aldehydes and other interfering agents. The probe 13 can be used for determining the MDA levels in mammalian cells and plant tissues.

**Fig. 13:**
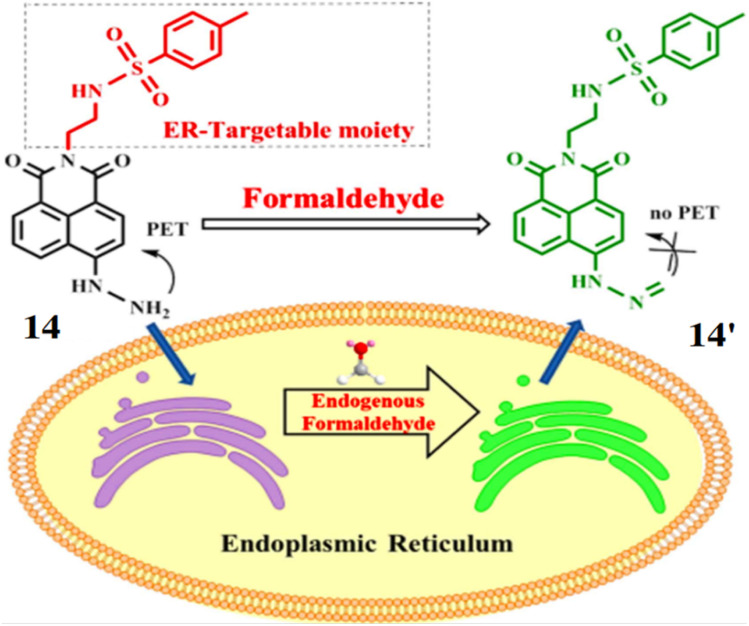
Chemical structure of 14 and the fluorescence response mechanism for detection of FA by 14 (reproduced from ref. 33 with permission from IOP Science, copyright 2017).

Tang *et al.* developed a probe 14 consisting of 1,8-naphthalimide as fluorophore and hydrazine as reactive site for the detection of formaldehyde.^33^ The hydrazine of 14 undergoes a condensation reaction with FA to give fluorescent compound 14′ showing absorption maximum at 440 nm and 140-fold increase in fluorescence at 541 nm with a detection limit at 5.24 × 10^−6^ M (Fig. 13). The probe 14 can be used for detection of endogenous FA in living cells due low cytotoxicity.

**Fig. 14:**
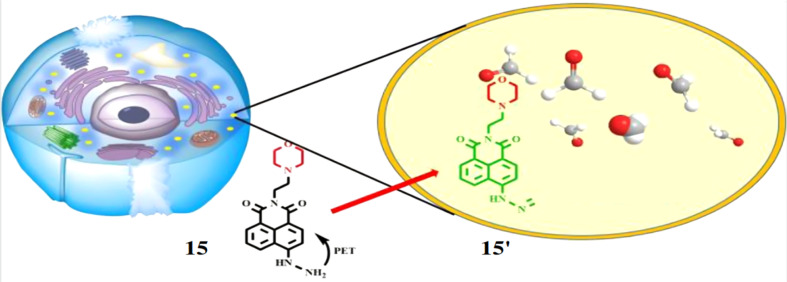
The fluorescence response mechanism of the lysosome-targetable 15 with FA (reproduced from ref. 34 with permission from American Chemical Society, copyright 2016).

Lin *et al.* developed a probe 15 for the detection of FA. The probe 15 consists of 1,8-naphthalimide chromophore, a hydrazine reaction site and morpholine, which acts as target site for lysosomes in cells.^34^ On reacting with FA, the probe 15 showed absorption maxima at 440 nm through 350-fold fluorescence enhancement by the formation of 15′ with a detection limit at 5.02 × 10^−6^ M (Fig. 14). The probe 15 is functional over 4.0–10.0 pH making it suitable for physiological pH and lysosomal pH levels and can be used for detection of FA in cells.

### 2.3 Detection by hydrazide

**Fig. 15:**
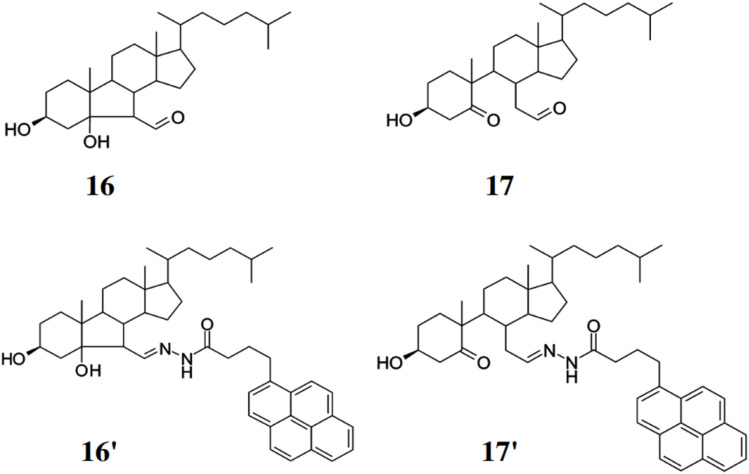
Structures of cholesterol carboxyaldehyde 16, cholesterol secocholestanal 17 and their corresponding fluorescent adducts with 1-pyrenebutyric hydrazide to form **16′** and **17′** (reproduced from ref. 35 with permission from American Chemical Society, copyright 2010).

Miyamoto *et al.* developed two fluorescent adducts 16′ and 17′ for the detection and quantification of cholesterol aldehydes comprising 1-pyrenebutyric hydrazine (PBH) with 3β-Hydroxy-5β-hydroxy-B-norcholestane-6β-carboxyaldehyde (16) generated by singlet molecular oxygen and 3β-Hydroxy-5-oxo-5,6-secocholestan-6-al (17) generated by ozone (Fig. 15).^35^ The adduct 16′ and 17′ can be detected using HPLC coupled with a fluorescent detector. Thus, preventing the formation of cholesterol aldehydes through Hock cleavage can help avoid overestimating the actual aldehyde concentration in the samples. Both the compounds 16 and 17 exhibited similar excitation and emission maxima wavelengths at 339 and 380 nm respectively. The detection limit for 16 was found out to be 10 fmol towards the formation of 16′. Thus, this method can be used to track and quantify cholesterol aldehydes under different oxidative conditions, which makes the method highly relevant for studying inflammation and oxidative stress-related diseases.

**Fig. 16:**
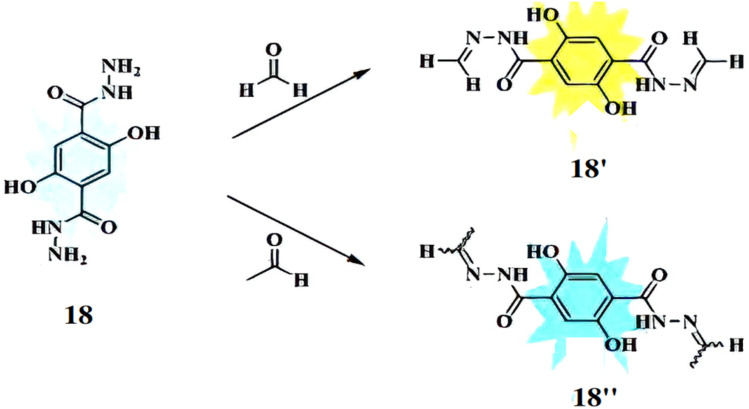
The mechanism of fluorescence changes for 18 reacting with formaldehyde **18′** and acetaldehyde **18′′** (reproduced from ref. 36 with permission from Elsevier, copyright 2020).

Guo *et al.* developed a fluorescent chemosensor, DTH (18), based on 2,5-dihydroxy-*p*-benzenedicarbonamide as the fluorophore and hydrazine as the reactive group, for distinguishing formaldehyde (FA) and acetaldehyde (AA).^36^ Probe 18 acts as a ratiometric sensor for FA (quantum yield: 0.055) and a turn-on sensor for AA (quantum yield: 0.122), forming distinct products (18′ and 18′′) with emission variations based on the FA/AA molar ratio (Fig. 16). It absorbs maximally at 355 nm and emits at 508 nm, with FA addition shifting the absorption to 400 nm and emission to 534 nm (Δ*λ* = 26 nm). For AA, absorption shifts to 392 nm with minor emission changes. The detection limits were 0.29 μM for FA and 0.26 μM for AA. Additionally, probe 18 can distinguish FA and AA in mixed solutions, is pH-sensitive, and is applicable for cell imaging and environmental analysis.

**Fig. 17:**
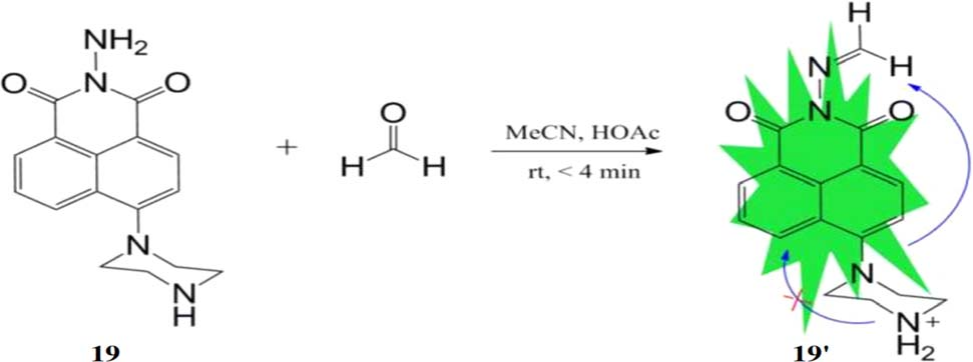
Structure of 19 and the reaction of hydrazine derivation with formaldehyde (reproduced from ref. 37 with permission from Elsevier, copyright 2016).

Lin *et al.* reported a probe, 2-amino-6-(piperazin-1-yl)-1H-benzo[de]isoquinoline-1,3(2H)-dione (19) for the detection of formaldehyde.^37^ The probe 19 reacts with formaldehyde in CH_3_CN (5% HOAc) at room temperature through an acylation reaction, forming a hydrazide structure (19′) with the enhancement of fluorescence intensity *via* ICT process (Fig. 17). The fluorescence intensity of 19 increases approximately by 13-fold in response to formaldehyde and about 6-fold with acetaldehyde. Probe 19 shows strong absorption at 256 nm and 382 nm, with a weak emission peak at 512 nm and a fluorescence quantum yield of 0.0073. The detection limit of 19 for formaldehyde is 0.25 ppm. The probe 19 can be incorporated into test papers, enabling quantitative detection of formaldehyde in both air and water.

## 3 Detection by cyclization reaction

**Fig. 18:**
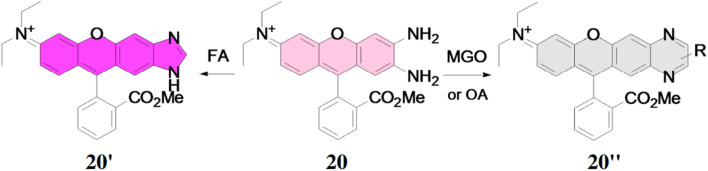
Structure of ligand 20 and its proposed reaction products with FA (formaldehyde), methylglyoxal (MGO) and oxalaldehyde (OA) (reproduced from ref. 38 with permission from Elsevier, copyright 2017).

Liu *et al.* described a probe 20 for detection of formaldehyde and methyglyoxal from other aldehydes *via* distinct emission patterns.^38^ The probe 20 is a derivative of *ortho*-diaminorhodamine and the detection is based upon the reaction kinetics between the probe and the analyte showing different fluorescence response for the reaction product. The probe 20 showed different reactive patterns for formaldehyde (on) and methylglyoxal (off) by a single wavelength excitation. The free probe 20 showed a weak fluorescence at 642 nm, but addition of formaldehyde caused strong fluorescence enhancement at 620 nm by the generation of 20′ and the addition of methylglyoxal resulted the decrease of at 620 nm by the formation of the product 20′′ (Fig. 18). The increase of fluorescence signal led to the enhancement of fluorescence quantum yield 0.07 to 0.55 with lower detection limit at 8.3 μM. Contrary to the previous, when the concentration of methylglyoxal was increased, fluorescence intensity was reduced with the decrease of fluorescence quantum yield from 0.07 to 0.014. Thus, probe 20 can be applied for the detection of formaldehyde, methylglyoxal and oxalaldehyde in living cells and also distinguish formaldehyde from the other two significantly.

**Fig. 19:**
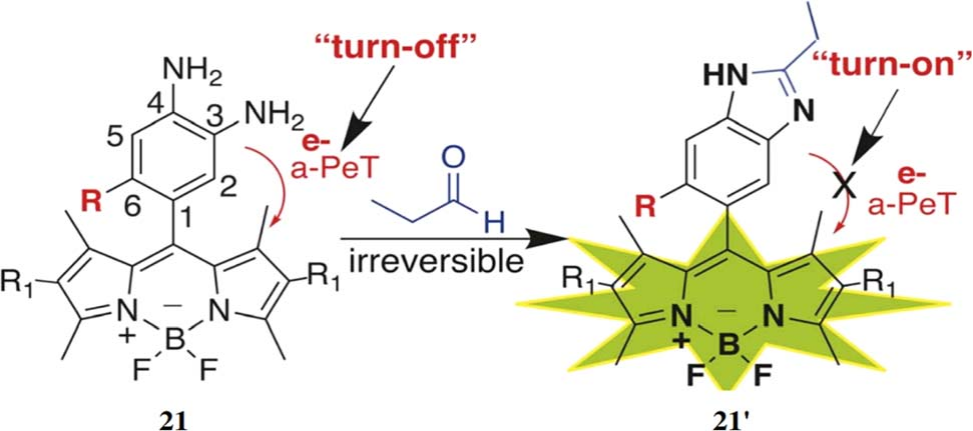
The reaction mechanism of BODIPY-diamine sensors 21 with propanal to generate turn-on benzimidazole products **21′** (reproduced from ref. 39 with permission from Royal Society of Chemistry, copyright 2023).

Raj *et al.* developed a sensor based on 3,4-phenyldiamine-BODIPY (21) for detecting small to long-chain aliphatic aldehydes.^39^ This sensor 21 reacts with aliphatic aldehydes irreversibly forming a benzimidazole derivative (21′), which produces a strong fluorescence enhancement by 26-fold at 507 nm with the increase of fluorescence quantum yield from 0.005 to 0.13 (Fig. 19). The limit of detection (LOD) was found out to be 2–10 μM inside live cells. The sensor 21 is phosphostable, non-cytotoxic and also able to permeate the cell membrane for the detection and monitor changes in aliphatic aldehydes in the living cells. The sensor 21 can be used to detect acetaldehyde levels in cancer cells and monitor aliphatic aldehyde levels in the presence of both ALDH2 activators and inhibitors.

**Fig. 20:**
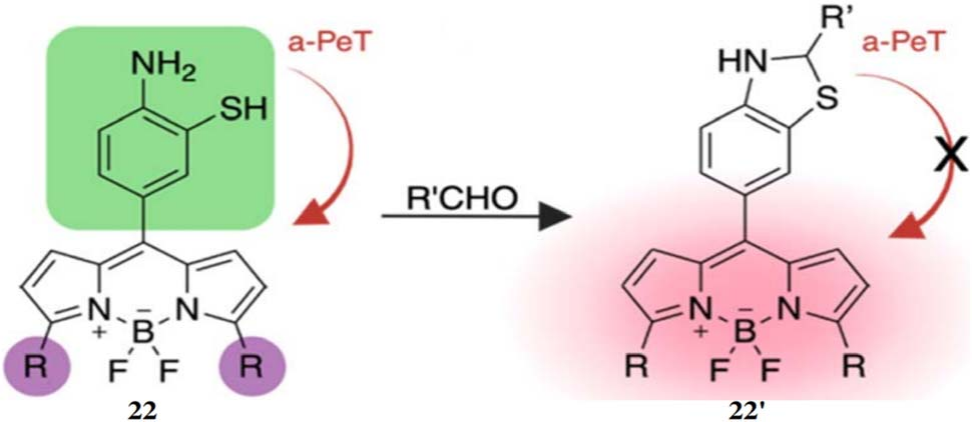
The chemical structure of probe 22 and probable sensing mechanism with aldehyde (reproduced from ref. 40 with permission from Royal Society of Chemistry, copyright 2024).

Raj *et al.* reported a BODIPY fluorophore probe for the detection of aldehydes, incorporating a bioorthogonal core of 2-aminothiophenol (22), which exhibits high selectivity toward aldehydes.^40^ Upon reacting with an aldehyde, the probe 22 generates dihydrobenzothiazole, producing fluorescence (22′) that can be tuned across the visible to near-infrared spectrum (Fig. 20). Probe 22 effectively detected both endogenous and exogenous total cellular aldehydes in live cells, organoids, and tissues. Its thiol group enhanced reactivity toward diverse aldehydes, including aromatic and α,β-unsaturated types, while significantly speeding up reaction kinetics. The probe 22 demonstrated a quantum yield of 0.009 and exhibited an 80-fold fluorescence increase after reacting with aldehydes. The maximum absorption (*λ*_max,abs_) and emission (*λ*_max,em_) wavelengths were determined to be 488 nm and 520 nm, respectively. The detection limit showcased a high dynamic range of 25–100 μM. This fluorescent probe 22 enables real-time recognition and quantification of aldehydes in live cells, tissues, organoids, and disease models, facilitating applications in disease diagnosis, drug screening, and studies of aldehyde-related biological processes.

**Fig. 21:**
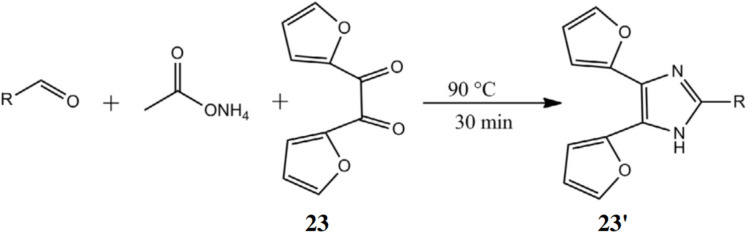
The reaction pathway of 23 with target aldehydes (reproduced from ref. 41 with permission from Elsevier, copyright 2014).

Kuroda *et al.* developed ligand 23 for a sensitive and accurate HPLC method with fluorescence detection to simultaneously determine lipoperoxidation-related aldehydes—glyoxal (GO), acrolein (ACR), malondialdehyde (MDA), and 4-hydroxy-2-nonenal (HNE)—in human serum.^41^ These aldehydes, generated during oxidative stress, can accumulate and cause cell death. Ligand 23 reacts with aldehydes to form fluorescent difurylimidazole derivatives (23′), detected with excitation at 250 nm and emission at 355 nm (Fig. 21). Detection limits ranged from 0.030 to 0.11 nmol mL^−1^, making probe 23 a useful tool for monitoring oxidative damage under various conditions.

**Fig. 22:**
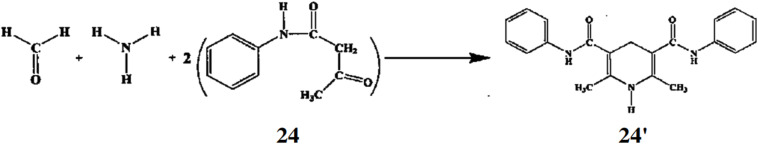
Chemical structure of acetoacetanilide (24) and the probable detection mechanism with formaldehyde (reproduced from ref. 42 with permission from Springer, copyright 2007).

Motomizu *et al.* presented acetoacetanilide (24) as ligand for the fluorometric estimation of formaldehyde based on the Hantzsch reaction.^42^ The probe 24 reacts with formaldehyde and ammonia to build an enamine-type intermediate, which undergoes cyclodehydration to yield a dihydropyridine backbone (24′) (Fig. 22). The maximum absorption wavelength is 368 nm, with excitation and emission maxima at 370 nm and 470 nm respectively. The detection limit of 24 towards formaldehyde was determined to be 2 × 10^−8^ M. The probe 24 was applied to determine formaldehyde in environmental water samples and demonstrated resistance to interference from various common substances in these samples, such as sodium chloride, acetone, and magnesium chloride.

**Fig. 23:**
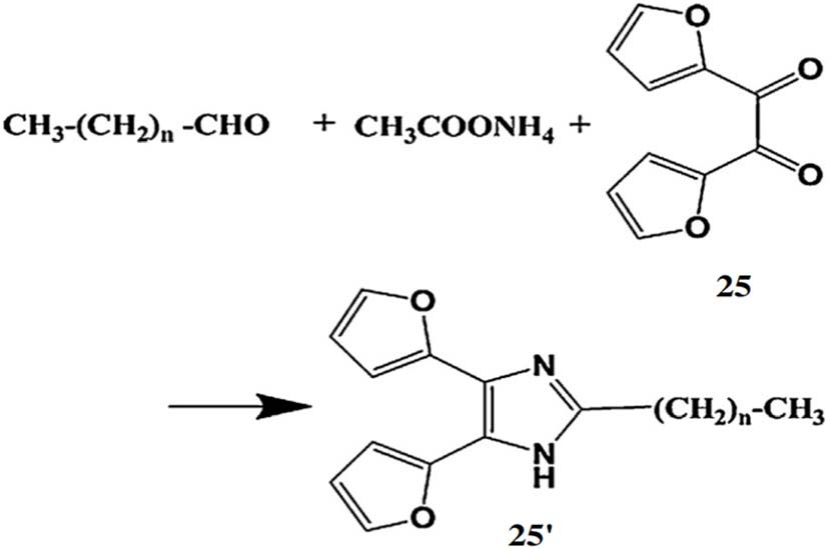
The detection pathway of 25 with various saturated aliphatic aldehydes (reproduced from ref. 43 with permission from Elsevier, copyright 2013).

Kuroda *et al.* developed a highly sensitive and selective fluorogenic probe, 2,2′-furil (25), for chromatographic determination of aliphatic aldehydes in human serum after pre-column derivatization.^43^ Probe 25 reacts with aldehydes in the presence of ammonium acetate to form fluorescent difurylimidazole derivatives (25′) (Fig. 23), which are detected by the emission signal at 355 nm upon excitation at 250 nm. The detection limits ranged from 0.19 to 0.50 nM. Probe 25 is effective for monitoring aliphatic aldehydes in human serum, including pentanal, hexanal, heptanal, octanal, nonanal, and decanal.

## 4 Detection by aza-Cope reaction

The aza-Cope rearrangement is a pericyclic reaction involving the [3,3]-sigmatropic rearrangement of an unsaturated iminium cation to generate an iminium ion intermediate. Aza-Cope has great modularity and generality and can be applied to fluorophores and luminophores to construct FA detecting and imaging probes. The aza-Cope rearrangement strategy provides enhanced selectivity and sensitivity for aldehyde detection compared to simple imine formation or cyclization. Unlike Schiff base formation, which is reversible and susceptible to hydrolysis or interference from other nucleophiles, the aza-Cope rearrangement leads to a more stable and distinct irreversible transformation.^44^

**Fig. 24:**
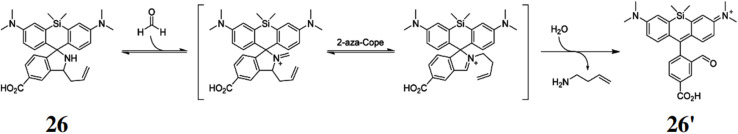
The structure of ligand 26 and the probable pathway for the formaldehyde sensing (reproduced from ref. 45 with permission from American Chemical Society, copyright 2015).

Chang *et al.* developed a fluorescence turn-on probe 26 for selective turn of fluorescence detection of formaldehyde by utilizing aza-Cope reaction transforming a homoallylic amine into an aldehyde with a fluorogenic turn-on response.^45^ Upon reaction of 26 with formaldehyde, imine formation followed by a 2-aza-Cope rearrangement and hydrolysis produces an aldehyde (26′) that cannot undergo spirocyclization, leading to a turn-on fluorescence (Fig. 24). Initially the probe 26 showed weakly fluorescence (*ε*_650_ = 190 M^−1^ cm^−1^, *Φ*_fl_ = 0.36) but shows a ∼8-fold emission enhancement (*λ*_max_ = 645 nm, *λ*_em_ = 662 nm) upon treatment with formaldehyde. The turn-on response was extended to ∼45-fold fluorescence enhancement with a detection limit of 5 μM. When the probe 26 was exposed to oxidizing and reducing conditions (H_2_O_2_ and glutathione) in the cells, there was no change in the fluorescence. Thus, probe 26 can be used to detect formaldehyde concentrations in aqueous buffers and live cells with high selectivity over other biological analytes.

**Fig. 25:**
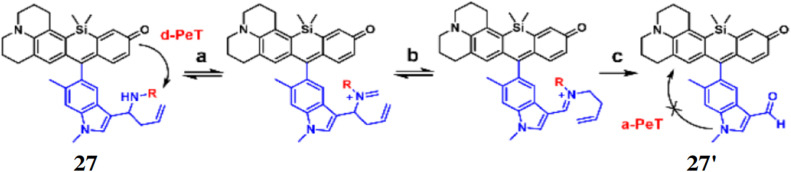
The formaldehyde detection strategy by the probe 27 utilizing 2-aza-Cope sigmatropic rearrangement. Labels a, b, and c represent condensation with formaldehyde, rearrangement, and hydrolysis steps, respectively (reproduced from ref. 46 with permission from American Chemical Society, copyright 2015).

Chan *et al.* reported a fluorescent probe 27 comprising julolidine-based silicon rhodol scaffold as the fluorescent core for the detection of formaldehyde through 2-aza-Cope sigmatropic rearrangement.^46^ The probe 27 features a homoallylic amine group, and a 4-nitrobenzyl moiety that acts as a dark quencher, suppressing fluorescence under non-reactive conditions. The amine group of 27 reacts with aldehydes to produce a fluorescent product, indole-3-carboxaldehyde (*Φ*_f_ = 0.11) (27′) (Fig. 25). The fluorophore exhibits an absorption maximum at 633 nm and emission in the range of 640–750 nm. Upon reacting with formaldehyde, the probe shows a 12.8-fold fluorescence enhancement, while with acetaldehyde, the fluorescence increases by 1.9-fold. The detection limit 27 with formaldehyde was calculated 0.01 mM. This probe 27 is suitable for studying formaldehyde-related processes in both normal and pathological conditions, such as neurodegenerative disorders where formaldehyde levels are elevated.

**Fig. 26:**
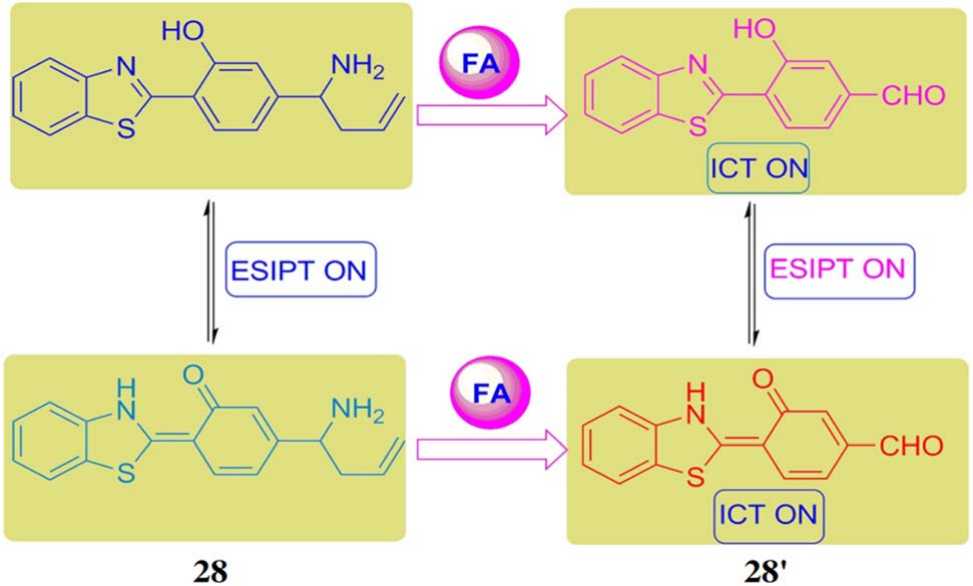
Chemical structure of 28 and reaction between 28 with formaldehyde (reproduced from ref. 47 with permission from Elsevier, copyright 2018).

Zheng *et al.* presented a ratiometric fluorescent probe 28 for the detection of formaldehyde based on the aza-Cope reaction.^47^ The probe 28 incorporates a 2-(2-hydroxyphenyl)benzothiazole scaffold, capable of undergoing intramolecular charge transfer upon reaction with formaldehyde. The ligand 28 also contains a pro-aza-Cope rearrangement group that reacts with formaldehyde (28′), causing a measurable red shift in fluorescence (Fig. 26). When excited at 350 nm, the probe exhibits an emission band at 462 nm, which shifts to 541 nm upon the addition of formaldehyde, accompanied by a 39-fold increase in emission ratios. The detection limit of 28 with formaldehyde was determined to be 4.1 × 10^−4^ M. This probe 28 is suitable for detecting formaldehyde in various media, including aqueous solutions, serum, and air.

**Fig. 27:**
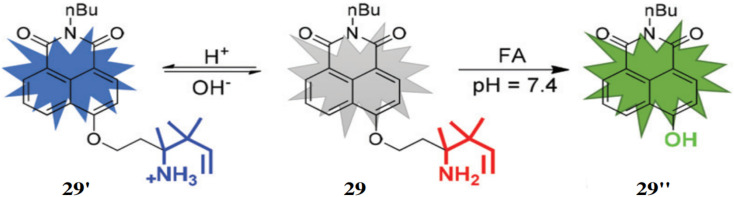
The chemical structure of 29 and the proposed mechanism for sensing of FA *via* aza-Cope arrangement (reproduced from ref. 48 with permission from Royal Society of Chemistry, copyright 2018).

Zhu *et al.* synthesized a dual function probe (29) for the detection of FA containing a 1,8-naphthalimide dye core and a homoallylamino group that triggers fluorescence changes depending on the environment.^48^ Under acidic conditions the probe 29 exhibits a blue fluorescence with an emission signal at 455 nm by the formation of 29′ and green fluorescence under basic conditions developing 29′′ with emission at 555 nm (Fig. 27). At low pH levels, probe 29 exhibited a 10-fold increase in fluorescence intensity at 455 nm. However, upon addition of formaldehyde (FA), the fluorescence intensity increased 22-fold, with the emission maximum shifting to 555 nm. The detection limit for FA was found to be 10 μM *in vitro* and 37 μM in living cells. Therefore, probe 29 can be used for simultaneous detection of pH and FA levels in living cells.

**Fig. 28:**
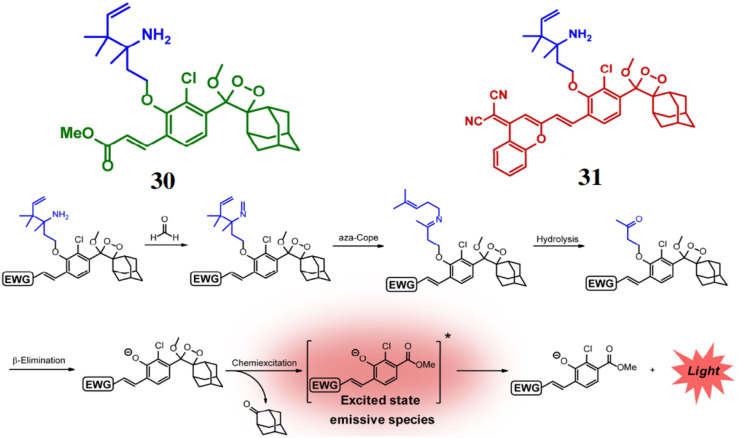
The structures of probes 30 and 31, and their reaction mechanism with FA (reproduced from ref. 49 with permission from Wiley-VCH, copyright 2018).

**Fig. 29:**
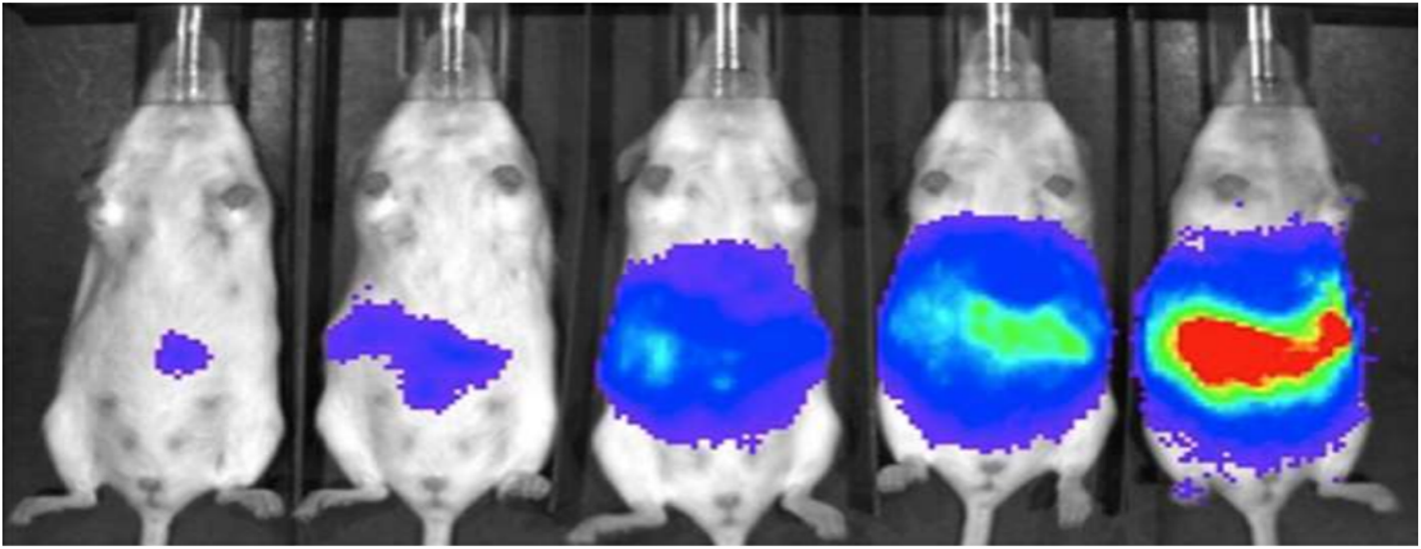
Chemiluminescent imaging of probe 31 in mice when injected with different doses of FA (0, 1.25, 2.5, 5, and 10 mg kg^−1^) (reproduced from ref. 49 with permission from Wiley-VCH, copyright 2018).

Chang *et al.* reported a pair of probes 30 and 31 for the detection of FA, based on a 2-aza-Cope reaction mechanism.^49^ Both the probes consist of Schaap's dioxetane scaffold which act the luminophore and electron-withdrawing groups to enhance the chemiluminescence efficiency. FA reacts with the homoallylamine group of both ligands, producing free phenoxy-dioxetane (Fig. 28). As a result, probe 30 exhibits an increase of emission intensity by 500-fold at 540 nm, while probe 31 shows a 33-fold increase at 700 nm. The detection limits for the probes were determined to be 10 μM for 30 and 25–50 μM for 31. Furthermore, the probe 30 can be used for detection of FA in cellular samples and 31 for live-animal FA visualization (Fig. 29).

**Fig. 30:**
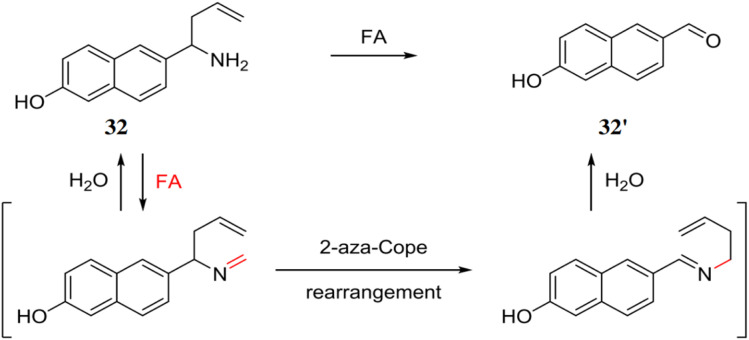
Chemical structure of the probe 32 and the proposed sensing mechanism of 32 with FA (reproduced from ref. 50 with permission from Elsevier, copyright 2016).

Sheng *et al.* synthesized a fluorescent probe 32 based on fluorophore 6-hydroxy-2-naphthaldehyde for the detection of FA through 2-aza-Cope rearrangement.^50^ The probe 32 reacts with FA to give highly fluorescent 32′ through 2-aza-Cope rearrangement exhibiting a strong fluorescence enhancement by 200-fold at 513 nm (Fig. 30). The probe 32 is highly selective to FA over other small molecules and has a detection limit of 0.57 μM. The probe 32 can be used for detection and quantification of FA in food samples, toffees and for FA imaging in HeLa cells.

## 5 Miscellaneous

**Fig. 31:**
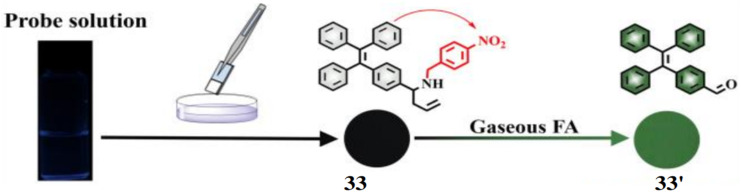
The preparation 33 loaded FA test plate and the fluorescence response to gaseous formaldehyde (reproduced from ref. 51 with permission from American Chemical Society, copyright 2018).

Yin *et al.* reported a fluorescent probe 33 incorporating a formaldehyde reactive moiety and fluorescence quencher (4-nitrobenzyl) into tetraphenylethylene (TPE) for the detection of gaseous formaldehyde.^51^ Initially, the chemosensor 33 exhibits weak fluorescence (*Φ*_FS_ = 2.64%) due to PET but upon reacting with gaseous formaldehyde, it showed highly emission (*Φ*_FS_ = 35.42%) by the formation of 33′ (Fig. 31). The fluorescence intensity of 33 in presence of formaldehyde test plates at 504 nm was increased by ∼8.7-fold in 60 minutes with a linear relation and the detection limit was found to be 0.036 mg m^−3^ which is lower than the air quality guideline value for gaseous formaldehyde (0.036 mg m^−3^) recommended by WHO. The changes in the fluorescence of the test plates can be observed by the naked eye under UV lamp and the formaldehyde test plates provide a safer and more convenient method for detection of gaseous formaldehyde in comparison to solution-based sensors.

**Fig. 32:**
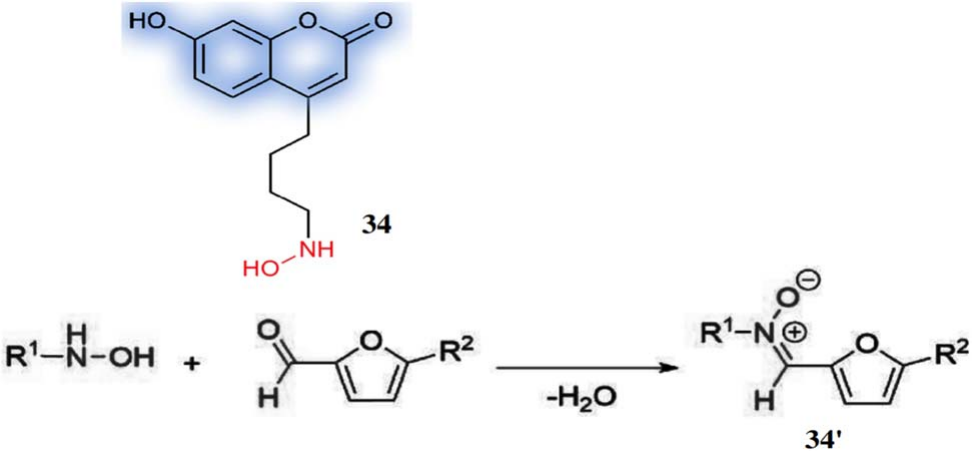
The structure of 34 and the nitrone formation pathway by the condensation 34 and furanic aldehyde to afford a nitrone derivative **34′** (reproduced from ref. 52 with permission from Elsevier, copyright 2017).

The probe 4-((hydroxyamino)butyl)-7-hydroxycoumarin (34) was developed by Hu *et al.* for the detection of various furfurals *e.g.* furfural (F), 5-methylfurfural (5-MF) and 5-hydroxymethylfurfural (5-HMF) based on nitrone formation.^52^ The probe 34 reacted with aldehyde group of furfurals forming a stable nitrone derivatives 34′ with a high fluorescence at 447 nm (*λ*_ex_ = 322 nm) and high quantum yield of 0.61 (Fig. 32). Significantly, the derivative (34′) achieved >10^4^ fold signal improvement than its underivatized counterpart and the detection limits ranges from 0.10 nM to 0.80 nM. The probe 34 can be utilized for the detection of furfurals in a variety of dried fruits, dairy products and also labeling of various aldehydes.

Summary of physiochemical properties, sensing mechanisms and applications of different chemosensors have been summarized in Table 1.

**Table 1:** Summary of physiochemical properties mechanistic view and probe type of different chemosensors

Sensors	*λ*_abs_ (nm)	*λ*_em_ (nm)	Detection limit (DL)	Photphysical processes/sensing mechanisms	Applications/advantages	References
1	296 nm	410 nm	0.003 nM	PET	Independent of pH	21
	368 nm					
2	488 nm	503 nm	—	PET	Selective fluorescence monitoring of aldehydes in organic solvents	22
3	548 nm	576 nm				
4		455 nm	16.6 μM	ESIPT	Distinguish aldehydes and ketone, detect 7 different aldehydes, and vapor detection	23
5	485 nm	538 nm	6.94 nM		Distinguish aldehydes from ketone, microbial oxidation monitoring	24
6	482 nm	525 nm	0.104 μM		Tracking of exo and endogenous formaldehyde in living cells and gaseous formaldehyde	25
7	670	708 nm	1.87 μmol L^−1^		Real-time detection of FA in food samples and endogenous FA in mice	26
8	—	550 nm	0.89 μg L^−1^	PET	Detection FA under strong acidic conditions	27
9	—	543 nm	7.1 × 10^−7^	PET	High selectivity for FA over other biological species, minimal toxicity, and photostability	28
10	450 nm	530 nm	—	PET	Ability to detect eight major oxidation products and seven aldehydes	29
11	—	FA: 550 nm	FA: 10.6 nM	Slow photon effect	Detection of FA and AA in air, aqueous and living systems	30
		AA: 553 nm	AA: 7.3 nM			
12	—	—	0.438 to 2.103 ppm	—	Suitable for detecting trace level of FA in living samples and atmosphere	31
13	373 nm	554 nm	4.54 × 10^−7^ M	PET	Functions in neutral and alkaline pH, compatible with physiological conditions and shows high selectivity for MDA in plant tissues and mammalian cells	32
14	440 nm	541 nm	5.24 × 10^−6^ M	PET	Detection of endogenous FA in living cells	33
15	440 nm	—	5.02 × 10^−6^ M	—	Suitable for physiological and lysosomal pH levels (4.0–10.0 pH)	34
16	339 nm	380 nm	10 fmol	—	Track and quantify cholesterol aldehydes	35
17						
18	355 nm	508 nm	FA: 0.29 μM	—	Distinguish FA and AA in mixed solution, pH sensitive and applicable for cell as well as environmental analysis	36
			AA: 0.26 μM			
19	256 nm	512 nm	0.25 ppm	ICT	Quantitative detection of FA in air and water	37
	382 nm					
20	—	642 nm	8.3 μM		Detection of FA, methylglyoxal, and oxalaldehyde, and distinguishing FA from the other two	38
21	—	507 nm	2–10 μM	PET	Detect AA levels in cancer cells and monitor aliphatic aldehyde levels in the presence of ALDH2 activators and inhibitors	39
22	488 nm	520 nm	25–100 μM	PET	Real-time recognition and quantification of aldehydes in live cells, tissues, organoids, and disease models	40
23	250 nm	355 nm	0.030 to 0.11 nmol mL^−1^	—	For monitoring oxidative damage under various conditions	41
24	368 nm	470 nm	2 × 10^−8^ M	—	Determine FA in water samples and resists interference from various substances	42
25	250 nm	355 nm	0.19 to 0.50 nM	—	Monitoring aliphatic aldehydes in human serum	43
26	645 nm	662 nm	5 μM	—	Detection of FA concentrations in aqueous buffers and live cells with high selectivity	45
27	633 nm	640–750 nm	0.01 mM	PET	Studying FA-related processes in normal and pathological conditions, like neurodegenerative disorders	46
28	350 nm	462 nm	4.1 × 10^−4^ M	ESIPT	Suitable for detecting FA in aqueous, serum and air	47
29	—	Acidic: 455 nm	10 μM *in vitro*		Simultaneous detection of pH and FA levels in living cells	48
		Basic: 555 nm	37 μM in living cell			
30	—	540 nm	10 μM	—	Detection of FA in cellular samples	49
31		700 nm	25–50 μM		Live-animal FA visualization	
32	—	513 nm	0.57 μM	—	Detection and quantification of FA in food samples, toffees and HeLa cells	50
33	—	504 nm	0.036 mg m^−3^	PET	Detection of gaseous FA by naked eye	51
34	322 nm	447 nm	0.10 nM to 0.80 nM		Detection of furfurals in a variety of dried fruits, dairy products and also labeling of various aldehydes	52

## 6 Conclusion

Aldehydes, particularly formaldehyde and acetaldehyde, pose significant environmental and health risks due to their widespread presence and adverse effects on human health. Conventional detection methods, such as gas and liquid chromatography, offer high accuracy but are often costly, complex, and less accessible, limiting their widespread use. In contrast, optical chemosensors based on fluorescence and colorimetric responses have emerged as promising alternatives, offering high sensitivity, selectivity, portability, and cost-effectiveness. Recent advancements in molecular design, utilizing mechanisms such as imine bond formation, cyclization reactions, and aza-Cope rearrangements, have significantly improved the performance of these sensors for aldehyde detection. Additionally, nanostructured assemblies have further enhanced their efficiency, enabling real-time and low-concentration monitoring in environmental, industrial, and biomedical applications. Despite these advancements, several challenges remain. Many sensors struggle with selectivity and sensitivity in complex real-world samples due to interference from other analytes, matrix effects, and limited stability. To overcome these limitations, future research should focus on developing universal sensing platforms with enhanced discrimination and minimal cross-reactivity. Integrating artificial intelligence for data processing, designing multi-analyte sensing arrays, and utilizing advanced nanomaterials could provide effective solutions. Furthermore, efforts should be directed toward miniaturizing sensor platforms and developing on-site analytical tools for practical, real-world applications. Addressing these challenges will be crucial for the widespread adoption of aldehyde sensors in environmental monitoring, food safety, and biomedical diagnostics, ultimately contributing to public health and environmental safety.

## Data availability

No primary research results, software or code have been included and no new data were generated or analysed as part of this review.

## Conflicts of interest

There are no conflicts of interest to declare.

## Abbreviations

FA: Formaldehyde
AA: Acetaldehyde
VOCs: Volatile organic compounds
WHO: The World Health Organization
OSHA: Occupational Safety and Health Administration
TMAB: 3,3′,5,5′-Tetramethyl-*N*-(9-anthrylmethyl)benzidine
PET: Photoinduced electron transfer
ESPIT: Excited-state intramolecular proton transfer
OPDA: *o*-Phenylenediamine
MDA: Malondialdehyde
ANH: [*N*-(3-*N*-Benzyl-*N*,*N*-dimethyl-propyl ammonium chloride)-1,8-naphthalimide]hydrazine
CNCs: Cellulose nanocrystals
PBH: 1-Pyrenebutyric hydrazine
LOD: Limit of detection
GO: Glyoxal
ACR: Acrolein
HNE: 4-Hydroxy-2-nonenal
TPE: Tetraphenylethylene
F: Furfural
5-MF: 5-Methylfurfural
5-HMF: 5-Hydroxymethylfurfural
